# Dr. Trotter, on Vaccine Inoculation

**Published:** 1800-06

**Authors:** T. Trotter

**Affiliations:** Cawsand Bay


### *To the Editors of the Medical and Phyſical Journal.*

GENTLEMEN,

FROM the animated and extenſive diſcuſſion which the ſubject of inoculation of the Vaccine diſeaſe, has undergone in your paſt Numbers, it appears that, in many inſtances, the firſt and often ſucceeding attempts have failed in ingrafting the *matter*. This diſappointment is the more to be lamented, as the patient may, in the interval between different trials, run the hazard of being expoſed to the natural variolous infection; and and thus bring the practice among designing or shallow observers, into disrepute, as it has frequently done the inoculation for Small-pox. Some of your correspondents, perhaps, impute this failure too much to the state of the vaccine matter; when they might find a better reason, from the particular condition of the skin, where the incision is made on the arm of the patient. The arm of the child is sometimes exposed for a considerable time, often while using persuasions to quiet his fears, or to hide his face from the sight of a lancet. If it happens to be cold weather, the skin becomes pale and flaccid, the blood and warmth retreat from the surface, and the sensibility of the spot is diminished; for the part of the arm which is usually cut, is not remarkably sensible. When my practice, formerly, lay much in this way; and finding my incisions often fail in communicating the variolous infection, particularly with very young children, I was in the habit of ordering the arm to be *well bathed* with warm milk and water; which, when wiped with a *rough towel*, would excite such a temporary inflammation on the spot, that I never failed afterwards. This gave the nerves of the part that susceptibility which was required to receive the poison; and I thought also, that the succeeding inflammation was quicker in consequence.

The JENNERIAN Inoculation has been introduced into this neighbourhood by Dr. Huggan, and earnestly supported by all the scientific part of the medical profession, as appears in your Journal. Like the early propagation of CHRISTIANITY, by its DIVINE LEADER, it was first "preached to the poor." * The children of poor soldiers and poor fishermen, first partook of its blessings: publicans and sinners have since embraced it; and the purity of its doctrine and practice is making proselytes to the very land's end in Cornwall. The time that the West Kent Regiment has been quartered in Plymouth, will therefore be a memorable period in the future History of Damnonia. But this is not the first instance where this regiment has been eminent for philanthropy. While quartered in Ireland, a dreadful infectious fever raged in the neighbourhood. These officers stepped forward; made their entertainments and amusements subservient to the wants of a starving multitude; opened, by their example, the purses of the rich; stemmed the torrent of contagion, and rescued hundreds from the grave. This heroic conduct convinced the deluded people in the district, that a regiment of British militia did not come for the purpose

* There has, as yet, been no attempt to render this inoculation general in the Navy.——Vid. Med. Nautica, Vol. II. Art, Small-Pox.

of dragooning them into submission, but to share with them the nobler attributes of British union, British feeling, and British charity. Such a fact as this does not belong exclusively to the ALBUM of Benevolence; it is strictly medical; and was its spirit general, there would be little occasion for disputations on *Nitrous Vapour or Fumigations.* While the age is frequently shocked with the horrid barbarities of parish officers, and views with regret the supine and reluctant trials which modern politicians have made to give parochial charity the pure practice of the British constitution, it is pleasing to hear, that it preserves its innate virtues among the officers of the army and the navy. I am,

GENTLEMEN,

Your obedient humble servant,

T. TROTTER.

